# Mesenchymal stem cell-derived angiogenin promotes primodial follicle survival and angiogenesis in transplanted human ovarian tissue

**DOI:** 10.1186/s12958-017-0235-8

**Published:** 2017-03-09

**Authors:** Yaoyao Zhang, Xi Xia, Jie Yan, Liying Yan, Cuilin Lu, Xiaohui Zhu, Tianren Wang, Tailang Yin, Rong Li, Hsun-Ming Chang, Jie Qiao

**Affiliations:** 1 Department of Obstetrics and Gynecology, Center for Reproductive Medicine, Peking University Third Hospital, No.49 North HuaYuan Road, HaiDian District, Beijing, 100191 China; 2 Beijing Key Laboratory of Reproductive Endocrinology and Assisted Reproduction, Beijing, 100191 China; 3 Key Laboratory of Assisted Reproduction, Ministry of Education, Beijing, 100191 China; 4 Department of Obstetrics and Gynecology, Center for Reproductive Medicine, Peking University Shenzhen Hospital, No.1120 Lotus Road, FuTian District, Shenzhen, Guangdong 518000 China; 5 Department of Obstetrics and Gynecology, Center for Reproductive Medicine, Shengjing Hospital of China Medical University, Shenyang, 100004 China; 6 Department of Obstetrics and Gynaecology, Child and Family Research Institute, University of British Columbia, Vancouver, V5Z4H4 Canada

**Keywords:** Ovarian tissue transplantation, Mesenchymal stem cell, Follicle survival, Fertility preservation, Angiogenin

## Abstract

**Background:**

We have recently reported that human bone marrow-derived mesenchymal stem cells (MSCs) facilitate angiogenesis and prevent follicle loss in xenografted human ovarian tissues. However, the mechanism underlying this effect remains to be elucidated. Thus, determining the paracrine profiles and identifying the key secreted factors in MSCs co-transplanted with ovarian grafts are essential for the future application of MSCs.

**Methods:**

In this study, we used cytokine microarrays to identify differentially expressed proteins associated with angiogenesis in frozen-thawed ovarian tissues co-transplanted with MSCs. The function of specific secreted factors in MSCs co-transplanted with human ovarian tissues was studied via targeted blockade with short-hairpin RNAi and the use of monoclonal neutralizing antibodies.

**Results:**

Our results showed that angiogenin (ANG) was one of the most robustly up-regulated proteins (among 42 protein we screened, 37 proteins were up-regulated). Notably, the targeted depletion of ANG with short-hairpin RNAi (shANG) or the addition of anti-ANG monoclonal neutralizing antibodies (ANG Ab) significantly reversed the MSC-stimulated angiogenesis, increased follicle numbers and protective effect on follicle apoptosis.

**Conclusion:**

Our results indicate that ANG plays a critical role in regulating angiogenesis and follicle survival in xenografted human ovarian tissues. Our findings provide important insights into the molecular mechanism by which MSCs promote angiogenesis and follicle survival in transplanted ovarian tissues, thus providing a theoretical basis for their further application.

**Electronic supplementary material:**

The online version of this article (doi:10.1186/s12958-017-0235-8) contains supplementary material, which is available to authorized users.

## Background

The transplantation of frozen-thawed ovarian tissue is a promising technique for the restoration of endocrine function and fertility [1], especially in cancer patients who have undergone gonadotoxic therapy [2–6]. However, ischemia-reperfusion injury and insufficient re-vascularization following transplantation, which lead to failure of follicular survival, are significant obstacles to the wider application of this technique [7–9]. Therefore, searching for methods to minimize this damage and to induce adequate revascularization is a critical step for successful ovarian tissue transplantation and the preservation of fertility.

Mesenchymal stem cells (MSCs), which can be isolated from various tissues [10], are a population of cells possessing pluripotent capabilities. MSCs have been shown to be a promising therapeutic approach to treat a spectrum of diseases, especially in disorders associated with insufficient angiogenesis [11, 12]. Recently, we demonstrated that MSCs from human bone marrow functioned as a supportive agent for human ovarian tissue transplantation, promoting angiogenesis and follicle survival [13]. However, the mechanism underlying this process is unknown. A better understanding of how co-transplanted MSCs exert their pro-angiogenic effects on ovarian grafts can help optimize the parameters for clinical applications. Therefore, in this study, we sought to investigate the possible mechanisms by which human MSCs promote re-vascularization and prevent follicle loss in xenografted ovarian tissues.

Angiogenesis is a multi-step process that involves the survival, proliferation, migration, and differentiation of endothelial cells, as well as tube formation and maturation. In recent years, several studies have used different approaches to investigate the mechanisms by which MSCs facilitate angiogenesis; however, no consistent conclusion has been reached. Some studies have indicated that MSCs are capable of differentiating into endothelial cells, pericytes, or even vessel walls to support the formation of blood vessels [14–16]. Other studies have suggested that MSCs are capable of protecting endothelial cells from apoptosis, including from oxidative stress-related apoptosis in the initial phase of angiogenesis [17]. Furthermore, in addition to the early steps of angiogenesis, such as endothelial cell proliferation, MSCs have been reported to support the late phases of angiogenesis, including blood vessel maturation [18, 19]. However, these proposed mechanistic explanations are still debated. Conflicting data have shown that morphological and ultrastructural evidence of MSC differentiation into endothelial cells or blood vessel wall structures in vivo is uncommon. In addition, the integration of the transplanted MSCs into host blood vessels has not been observed in animal studies [20].

In the past several years, research has focused on the secretion-based paracrine regulatory role of MSCs. Studies have shown that MSCs can establish a pro-angiogenic microenvironment by persistently secreting bioactive molecules that promote angiogenesis and microvascular network formation [21]. Furthermore, accumulating reports have suggested that the therapeutic potential of MSCs is largely dependent on their secretory capacity rather than on their differentiation capacity [22]. Studies of MSC secretion profiles have shown that some pro-angiogenic cytokines, such as VEGF, MCP-1 and FGF-2, can be detected in the conditioned medium of MSCs [23]. However, the composition of the profile of secreted molecules under different conditions varies greatly, leading to differences in angiogenic capacities [24]. For example, it has been reported that under hypoxic conditions, the secretion of pro-angiogenic factors can be significantly increased [25]. Due to these variations and the limited knowledge regarding the cytokine production of MSCs following transplantation (a hypoxic condition), determining the paracrine profiles and identifying the key secreted cytokines in MSCs co-transplanted with ovarian tissues are essential steps before further (pre)clinical studies can be performed. Therefore, in the present study, we designed an antibody-based cytokine microarray to investigate the expression profiles of angiogenesis-related proteins in frozen-thawed ovarian tissues co-transplanted with MSCs. We found that a panel of proteins, including angiogenin (ANG), was significantly up-regulated. Hence, we sought to suppress ANG secretion in MSCs via RNAi with an ANG-specific short-hairpin RNA (shANG) or using ANG monoclonal antibodies to explore the role of ANG in angiogenesis and follicle survival induced by MSCs in ovarian transplantation.

## Methods

### Identification and isolation of human MSCs

The protocols for the collection of human MSCs and ovarian samples were approved by the Ethics Committee of the Peking University Third Hospital. Informed consent was obtained from all subjects. Human MSCs were isolated by density gradient centrifugation (Additional file 1: Figure S1). The detailed methods were described previously [26]. Human MSCs were isolated from the bone marrow of a healthy female (age: 28 years old) who underwent bone marrow harvesting for allogeneic bone marrow transplantation at our hospital. Human MSCs were cultured in alpha minimum essential Eagle’s medium (α-MEM) supplemented with 20% human serum albumin (Life Technologies, Carlsbad, CA), L-glutamine and penicillin-streptomycin (Invitrogen, Life Technologies, Grand Island, NY) and used for experiments during passages 3 to 8. For identification, the MSCs were stained with antibodies against CD34, CD44, CD45, CD19, CD90, and CD105 (Biolegend, San Diego, CA). The detail information about surface marker selection and stemness potential characterization of human MSCs were provided previously [26].

### Lentivirus production and infection

An ANG-specific short-hairpin RNAi (shANG) was synthesized and cloned into a lentiviral GV248 vector (Genechem, China). The sequences for RNAi were designed to knock down the gene expression of ANG (shANG) or were control particles (shCTRL) and were obtained using the shRNA library of the RNAi Codex (www.codex.cshl.edu).

The target sequences were as follows: shANG: 5′- CCACTTGGATCAGTCAATT -3′ and shCTRL: 5′-AACAGTCGCGTTTGCTACTTT-3′. Lentivirus preparation, infection and selection were performed according to the technical manual for the vector.

### Quantitative real-time PCR and ELISA

Total RNA was extracted from MSCs transfected with shANG or shCTRL using a Total RNA Purification Plus Kit (Norgen, Canada), according to the manufacturer’s instructions. A total of 1.0 μg of RNA was reverse-transcribed using the SuperScript III First-strand Synthesis System. PCR amplification was carried out in an ABI 7500 Detection System using Power SYBR (Applied Biosystems, Carlsbad, CA) under the following conditions: 50 °C for 2 min; 95 °C for 2 min; and 40 cycles of 95 °C for 15 s, 60 °C for 15 sec, and 72 °C for 1 min. GAPDH was used as a reference gene for normalization. The level of *ANG* mRNA expression was calculated using the following formula: 2^(ΔCt Test – ΔCt Control)^. The *Ct* of *ANG* was compared with that of the internal control GAPDH gene.

The primer sequences used for PCR were as follows:

GAPDH sense: 5′- TGACTTCAACAGCGACACCCA -3′ and antisense: 5′- CACCCTGTTGCTGTAGCCAAA -3′; ANG sense: 5′- CCTCCATGCCAGTACCGAG -3′ and antisense: 5′- GGACGACGGAAAATTGACTGA -3′.

We used an ELISA kit (R&D, Abingdon, UK) for the quantitative measurement of human ANG in the conditioned media of MSCs transfected with specific shANG or shCTRL after 24 h of culture. MSCs were plated on a 6-well plate at a density of 10^5^ cells/well. ELISAs were performed according to the manufacturer’s instructions. Each sample was analyzed in triplicate.

### Collection and treatment of human ovarian tissue

The use of human ovarian tissues was reviewed and approved by the ethics committee of Peking University (registration number: 2009005). Human ovarian tissue was obtained from a 26-year-old female patient who underwent gender reassignment surgery. One biopsy from each ovary was obtained and cut into small pieces after removing the medulla tissues. The ovarian tissues were cryopreserved and thawed as previously described [26]. Briefly, the ovarian tissue was transported from the operating room to the laboratory in Leibovitz’s L-15 medium (Invitrogen, Carlsbad, CA) supplemented with 1% human serum albumin (Life Technologies, Carlsbad, CA), 100 IU/mL penicillin (Sigma, St. Louis, MO) and 100 μg/mL streptomycin (Sigma, St. Louis, MO). After enucleating the medulla with surgical scissors and a scalpel, the ovarian cortical tissues were manually cut into small pieces with a size of 5 mm × 5 mm × 1 mm (thickness).

Two slices of ovarian cortical tissues were placed in a 1.8-mL cryovial (Nunc, Roskilde, Denmark) containing 1 mL of 1.5 mol/L DMSO (Sigma-Aldrich, St. Louis, MO), 0.1 mol/L sucrose (Sigma-Aldrich, St. Louis, MO) and 10% HSA (Life Technologies, Carlsbad, CA) in Leibovitz medium. After 30 min of exposure to the cryoprotective agent at 4 °C, the cryovial was transferred to a program freezer (Biomed Freezer Kryo 10, series II; Planer, Middlesex, UK). The freezing and thawing procedures were carried out according to the techniques described by Andersen [27].

### Ovarian transplantation in severe combined immune deficiency mice

All animal procedures were approved by the Institutional Animal Care and Use Committee of the Peking University Third Hospital. A total of 30 8-week-old female ovariectomized mice with severe combined immune deficiency (SCID) (Animal Center of Medical College of Peking University) were used in the study. Consistently with previous reports [13, 28, 29], each ovarian fragment was co-transplanted with 5 × 10^5^ MSCs in our study,.

For the array analysis, 6 mice were randomly assigned to one of 2 groups: (1) Graft + MSC group: each ovarian fragment was transplanted with 5 × 10^5^ MSCs packaged in 10 μL of Matrigel (Corning, USA); and (2) Graft group (control): each ovarian fragment was transplanted in 10 μL of Matrigel. The grafts were retrieved and rapidly frozen in liquid nitrogen for cytokine array analyses 7 days after transplantation.

For the shRNA blockade experiment, 12 mice were divided into 4 equal groups: (1) Graft group: each ovarian fragment was transplanted with 10 μL of Matrigel; (2) Graft + MSC group: each ovarian fragment was transplanted with 5 × 10^5^ MSCs packaged in 10 μL of Matrigel; (3) Graft + shCTRL MSC group: each ovarian fragment was transplanted with 5 × 10^5^ shCTRL- transfected MSCs packaged in 10 μL of Matrigel; and (4) Graft + shANG MSC group: each ovarian fragment was transplanted with 5 × 10^5^ stably shANG-transfected MSCs packaged in 10 μL of Matrigel.

For the antibody blockade experiment, 12 mice were divided into 4 equal groups:

(1) Graft group: transplantation of ovarian tissues with 10 μL of Matrigel; (2) Graft + MSC group: each ovarian fragment was transplanted with 5 × 10^5^ MSCs packaged in 10 μL of Matrigel; (3) Graft + MSC + Control antibody (Ab) group: each ovarian fragment was transplanted with 5 × 10^5^ MSCs with 200 μg/mL of a control isotype IgG antibody (Thermo Fisher Scientific, Carlsbad, CA) packaged in 10 μL of Matrigel; and (4) Graft + MSC + ANG Ab group: each ovarian fragment was transplanted with 5 × 10^5^ MSCs with 200 μg/mL of the ANG monoclonal antibody 26-2 F (Millipore, Germany) packaged in 10 μL of Matrigel.

The detailed transplantation procedure has been described previously [13]. Briefly, before transplantation, the SCID mice were anesthetized via intraperitoneal injection of 2,2,2-tribromoethyl alcohol (Sigma, St. Louis, MO) and tertamyl alcohol (Sigma, St. Louis, MO). Two skin incisions were made in the lower third of the abdominal wall on both sides after sterilizing the area. The ovarian tissues were randomly chosen and xenografted into the subcutaneous interspace, where the cortical side adhered to the skin and the medullar side adhered to the fascia. Two pieces of ovarian tissue were then placed in each mouse. The skin incisions were subsequently closed with absorbable 5/0 Prolene (Ethicon, Somerville, NJ). All procedures were performed under aseptic conditions. The animals were euthanized via cervical dislocation after 7 days (3 mice in each group). Ovarian cortical tissues were retrieved and fixed in 4% formaldehyde for histological and immunohistochemical examination.

### Human angiogenesis array

A human antibody-based array kit was purchased from Raybiotech (Raybiotech Inc, Norcross GA); in the kit, glass slides were printed as sub-arrays consisting of 42 antigen-specific antibodies against angiogenesis-related factors. The antibody array was used according to the manufacturer’s instructions. The results were normalized to internal positive controls for comparison. Two replicates per antibody were spotted, and the average of the median signal intensity from each spot (minus local background subtraction) was used for the calculation. The arrays were visualized using ImageQuant LAS4000 software (GE Healthcare) and analyzed using ImageJ (National Institutes of Health).

### Assessment of ovarian histology

Ovarian histology was evaluated based on hematoxylin and eosin (HE) staining. The detailed procedures have been described previously [13]. After fixation, xenografted ovarian tissues were processed for routine paraffin embedding, and 5-μm-thick serial sections were prepared. One from every five serial sections was used for HE staining, and follicles were counted in five randomly selected fields at × 400 magnification. To avoid counting follicles more than once, only follicles with a visible nucleus were counted. Follicle stages were classified as previously described [30]. The number of primordial follicles in each group was expressed as the sum of the follicles in 25 different sections.

### Immunohistochemistry and Immunofluorescence study

Immunohistochemistry was performed using the ABC Staining System (Zhongshan Golden Bridge Biotechnology, Inc., Beijing, China). The detailed procedures have been described previously [31]. For CD-31 staining, human fetal villus tissues (10-week-old) were used as a positive control. For AC-3 staining, thawed human ovaries (female, age: 21) were used as a positive control. The primary antibody was omitted in the negative control. The follicles with positive staining (brown staining in the cytoplasm/nucleus) and the total number of follicles were counted in five random fields (×400) of each section in 25 different sections in each sample. To evaluate vascular density, CD31-positive vessels were counted in five random fields (×400 magnification) of each section and in 5 different sections from each sample. Immunofluorescence staining of CD31 and Ki67 were performed as described previously [32].

### Statistical analysis

All experiments were repeated three times unless specified otherwise. Analyses were performed using SPSS 13.0 software (SPSS, Inc., Chicago, IL, USA). For the microarray, qPCR and ELISA data, statistical significance was determined with a two-tailed Student’s *t* test. The vascular density, follicle count and AC-3 percentage data were analyzed via one-way or two-way analysis of variance (ANOVA), followed by pairwise comparisons using the LSD method. All *P* values were two-sided. A *P* value < 0.05 was considered statistically significant.

## Results

### ANG levels are significantly increased upon co-transplantation of MSCs with ovarian tissues

**Fig 1:**
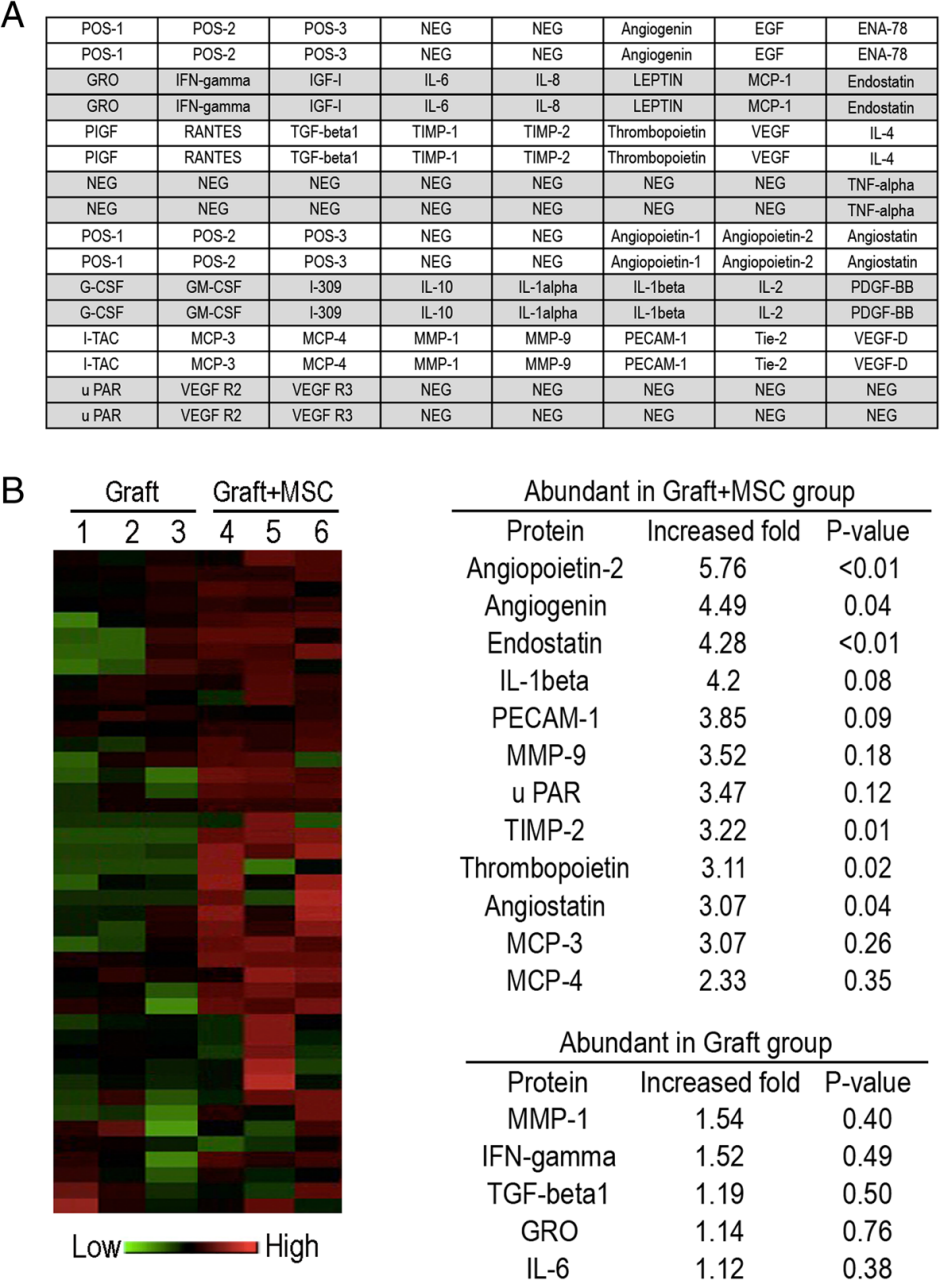
Angiogenin level is significantly increased in co-transplanted of ovarian tissues and MSCs. **a** List of antibodies against angiogenesis related cytokine factors by RayBiotech human antibody array; **b** Comparison of the panel of protein expression profiles in ovarian graft and MSCs co-transplantation (Graft + MSC group) versus Graft group. (*Left*) Heat maps were developed with the hierarchical clustering algorithm between the two groups. For each protein, signals from all conditions were averaged to generate the baseline. Signals above baseline are red; signals below baseline are green. The heat map key shows log2-fold changes from baseline. (*Right*) Lists of differential expressed protein that was abundant in Graft + MSC group or in MSC group. MSC, mesenchymal stem cells. POS, positive control. NEG, negative control

The characterization of MSCs was shown in Additional file 2: Figure S2. The isolated MSCs were negative for CD34, CD45, and CD19 and positive for CD44, CD90, and CD105. IHC study showed scattered positive expression of CD90 and CD105 in the ovarian tissue co-transplanted with MSCs (Additional file 3: Figure S3). To investigate whether certain secreted factors were specifically increased in the co-transplanted ovarian tissues and MSCs, we analyzed 42 angiogenesis-related proteins via protein microarray analysis (Fig. 1). The results were compared with the Graft group (xenografted ovarian tissues only). In total, the expression levels of 37 proteins were higher in the Graft + MSC group, whereas the expression levels of 5 proteins were higher in the Graft group. Twelve proteins were at least two-fold more abundant in co-transplanted ovarian tissue and MSCs than in solely ovarian grafts (Fig. 1b). The remaining proteins exhibited non-significant changes. By contrast, no proteins were at least twice as abundant in solely ovarian grafts as in co-transplanted ovarian tissue and MSCs. We were intrigued that ANG exhibited a marked increase in expression (up to 4.49-fold) in co-transplanted ovarian tissue and MSCs, particularly as it has been reported that ANG plays an essential role in endothelial cell proliferation and angiogenesis [33]. Although the expression of angiopoietin-2 was even higher (by up to 5.76-fold), angiopoietin-2 has been associated with endothelial cell death and vascular regression [34]. Therefore, we focused on ANG in our subsequent experiments.

### Blockade of ANG suppresses MSC-stimulated ovarian angiogenesis

Because ANG was robustly increased in MSCs co-transplanted with ovarian grafts, we speculated that ANG might play a key role in angiogenesis and follicle survival in MSCs co-transplanted with ovarian grafts. To test our hypothesis, we specifically inhibited ANG function in MSCs using either shANG or an anti-ANG neutralizing antibody. The results of RT-PCR indicated that shANG significantly suppressed the mRNA expression of ANG, with a 72.4% knockdown efficiency (*P* < 0.01, Additional file 4: Figure S4A). In addition, secreted ANG was measured in the cell culture media using a commercial ELISA. As expected, a significantly lower secreted ANG level was observed in the shANG-transfected MSCs than in the shCTRL-transfected MSCs (*P* < 0.01, Additional file 4: Figure S4B).

**Fig 2:**
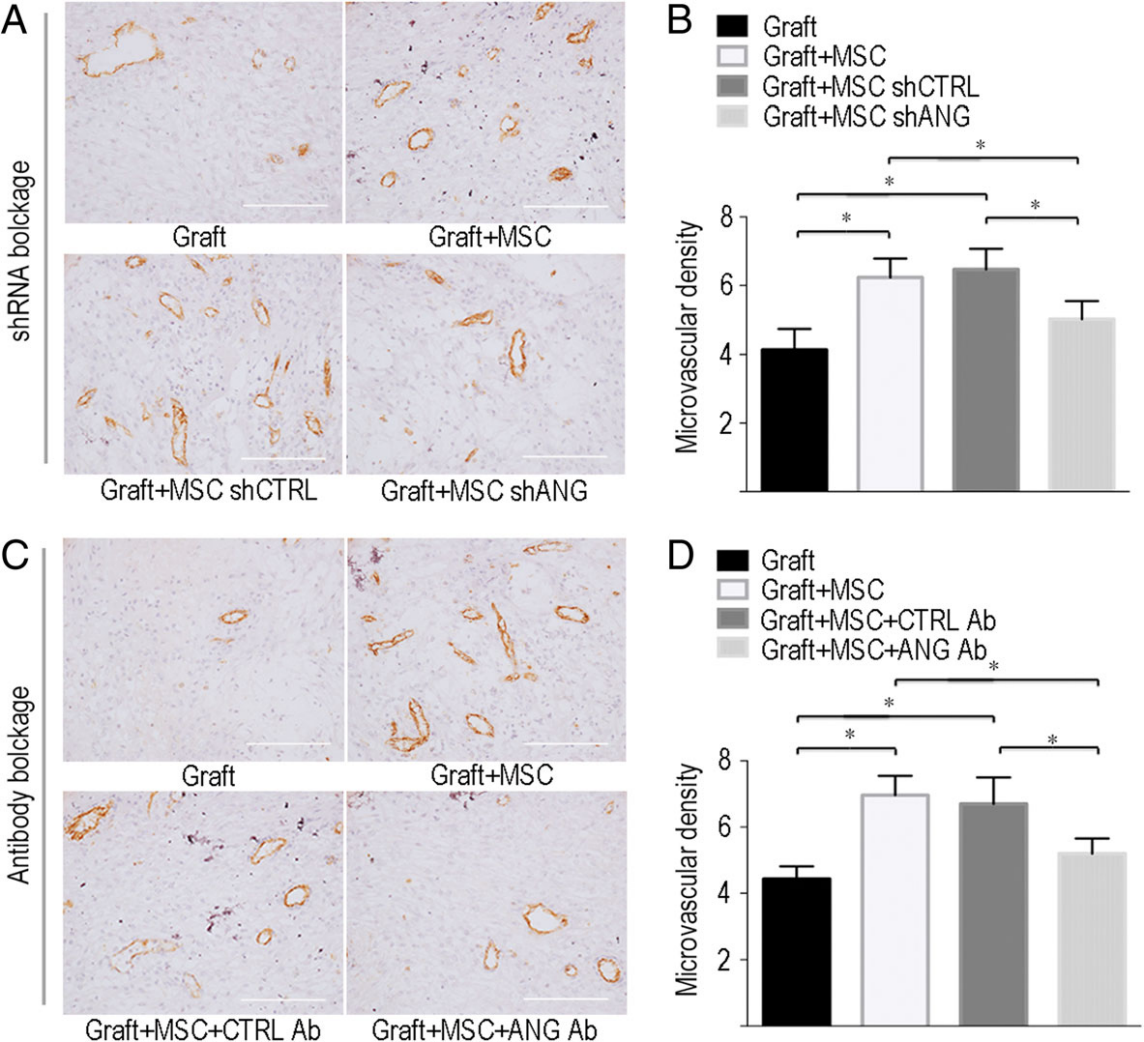
Angiogenin-blockade suppressed MSCs-stimulated ovarian angiogenesis. **a** Representative figures showing microvascular density of ovarian tissues in shRNA blockage analysis, as determined by CD31 immunoactivity, at post-transplantation day-7 in each group. **b** Quantitative analysis of microvascular densities between different groups in shRNA blockage analysis, **c** Representative figures showing microvascular density of ovarian tissues in antibody blockage experiment, as determined by CD31 immunoactivity, at post-transplantation day-7 in each groups. **d** Quantitative analysis of microvascular density between different groups in antibody blockage experiment. Data shown are means ± SEM of triplicates in a representative experiment. MSC, mesenchymal stem cells; ANG, angiogenin; CTRL, control; Ab, antibody; shANG, ANG specific short hairpin RNA; shCTRL, control short hairpin RNA, * *P* < 0.05. Scale bar = 100 μm

Ovarian angiogenesis in each group in the shRNA and antibody blockade experiments was assessed via CD31 staining and microvascular density counts (Fig. 2a, c). Similar to our previous study, by day 7, co-transplantation of MSCs with the graft (Graft + MSC group) had significantly promoted the density of CD31-positive microvessels compared with the densities in the Graft group (Additional file 5: Figure S5) in both the shRNA (6.23 ± 0.55 vs. 4.13 ± 0.61, respectively, *P* < 0.05, Fig. 2b) and ANG antibody blockade experiments (6.97 ± 0.59 vs. 4.43 ± 0.38, respectively, *P* < 0.05, Fig. 2d). In the shRNA blockade experiment, the microvascular density was significantly decreased in the Graft + MSC shANG group (5.03 ± 0.51) compared with those of the Graft + MSC (6.23 ± 0.55, *P* < 0.05, Fig. 2b) and Graft + MSC shCTRL groups (6.47 ± 0.60, *P* < 0.05, Fig. 2b). This finding showed that ovarian angiogenesis induced by MSCs was significantly suppressed by shANG, most likely due to the suppression of ANG secretion. Although shCTRL did not suppress MSC-induced angiogenesis, the microvascular density in the Graft + MSC shCTRL groups (6.47 ± 0.60) was not significantly different from that in the Graft + MSC groups (6.23 ± 0.55, *P* = 0.63, Fig. 2b). In the antibody blockade experiment, we found that the microvascular density was significantly decreased in the Graft + MSC + ANG Ab group (5.2 ± 0.46) compared with those in the Graft + MSC group (6.97 ± 0.59, *P* < 0.05, Fig. 2d) and the Graft + MSC + CTRL Ab group (6.70 ± 0.80, *P* < 0.05, Fig. 2d), which showed that the ANG Ab sufficiently blocked MSC-induced angiogenesis, probably via the neutralization of ANG secretion. By contrast, the CTRL IgG Ab had no influence on the effect of MSCs on ovarian angiogenesis, as the microvascular density in the Graft + MSC + CTRL Ab group (6.70 ± 0.80) was not significantly different from that in the Graft + MSC group (6.97 ± 0.59, *P* = 0.45, Fig. 2d).

### Blockade of ANG neutralizes MSC-stimulated follicular survival

**Fig 3:**
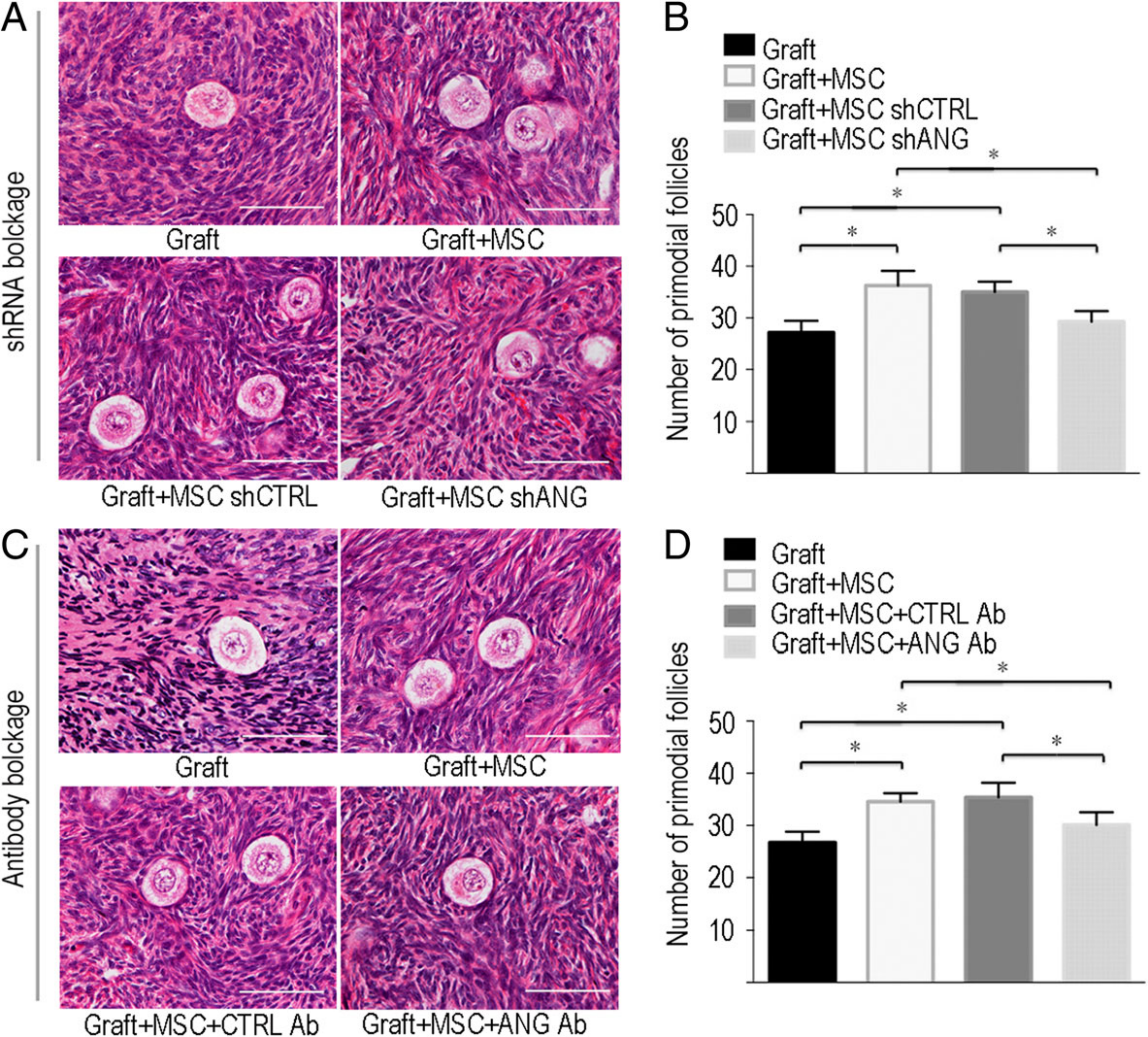
Blockade of angiogenin neutralized MSC-stimulated follicular survival after co-transplantation of MSCs. **a** Representative images of HE-stained section showing number of primodial follicles in ovarian tissue at post-transplant day-7 of each group in the shANG blockage experiment. **b** Quantitative analysis of follicular numbers in different groups in shANG blockage experiment. **c** Representative images of HE-stained section showing number of primodial follicles in ovarian tissue at post-transplant day-7 of each group in antibody blockage experiment. **d** Quantitative analysis of primodial follicular numbers in different groups in antibody blockage experiment. Data shown are means ± SEM of triplicates in a representative experiment. MSC, mesenchymal stem cells; ANG, angiogenin; CTRL, control; Ab, antibody; shANG, ANG specific short hairpin RNA; shCTRL, control short hairpin RNA, * *P* < 0.05. Scale bar = 50 μm

Before transplantation, the average number of primodial follicles in ovarian tissue is 63.17 ± 9.52. Next we examined the number of primordial follicles in xenografted ovarian tissues after ANG blockade in MSCs using either shRNA or a neutralizing antibody. Representative HE-stained follicles from each group are shown in Fig. 3 and Additional file 6: Figure S6. Consistent with our previous study, the co-transplantation of MSCs with ovarian tissues (Graft + MSC group) significantly increased the follicle count compared with that of the Graft group in both the shRNA (36.27 ± 2.85 vs. 27.13 ± 2.32, respectively, *P* < 0.05, Fig. 3b) and antibody blockade experiments (34.57 ± 1.60 vs. 26.73 ± 2.07, respectively, *P* < 0.05, Fig. 3d). In the shRNA experiment, the numbers of primordial follicles in the Graft + MSC shANG group (29.27 ± 2.05) were significantly lower than those in the Graft + MSC (36.27 ± 2.85, *P* < 0.05, Fig. 3b) and the Graft + MSC shCTRL groups (35.03 ± 1.99, *P* < 0.05, Fig. 3b). This result indicates that the suppression of ANG secretion by shANG significantly neutralized the ability of MSCs to enhance follicular survival. However, shCTRL did not attenuate the effect of MSCs on the enhancement of follicular survival, as the numbers of primordial follicles in the Graft + MSC + CTRL Ab group (35.03 ± 1.99) was not significantly different from that in the Graft + MSC group (36.27 ± 2.85, Fig. 3b). In the antibody blockade experiment, the number of primordial follicles in the Graft + MSC + ANG Ab group (30.10 ± 2.43) was significantly lower than those in the Graft + MSC (34.57 ± 1.60, *P* < 0.05, Fig. 3d) and Graft + MSC + CTRL Ab groups (35.40 ± 2.78, *P* < 0.05, Fig. 3d), indicating that the suppression of ANG secretion by the ANG antibody significantly neutralized the ability of MSCs to enhance follicular survival. However, the CTRL IgG Ab did not have a neutralizing effect, as the number of primordial follicles in the Graft + MSC + CTRL Ab groups (35.40 ± 2.78) was not significantly different from that in the Graft + MSC group (34.57 ± 1.60, *P* = 0.66, Fig. 3d).

### Blockade of ANG increases follicular apoptosis after the co-transplantation of MSCs

**Fig 4:**
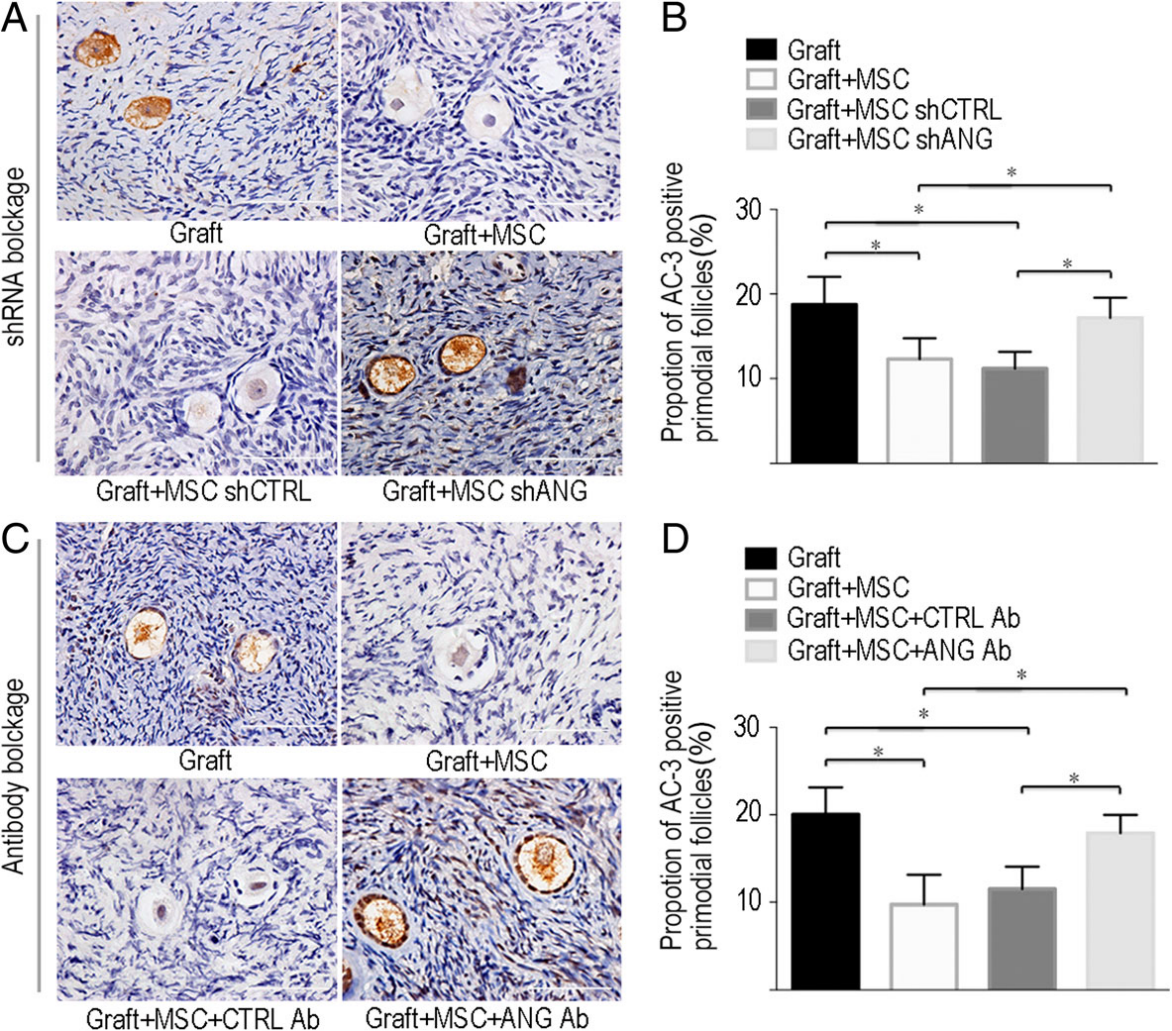
Blockade of angiogenin increased primordial follicular apoptosis after co-transplantation of MSC. **a** Representative images showing primordial follicular apoptosis as determined by AC3 staining of ovarian tissues at post-transplantation day-7 in different groups in shANG blockage experiment. **b** Quantitative analysis of primordial follicular apoptosis between different groups in shANG blockage experiment. **c** Representative images showing primordial follicular apoptosis as determined by AC3 staining of ovarian tissues at post-transplantation day-7 in different groups in antibody blockage experiment. **d** Quantitative analysis of primordial follicular apoptosis between different groups in antibody blockage experiment. Data shown are means ± SEM of triplicates in a representative experiment. MSC, mesenchymal stem cells; ANG, angiogenin; Ab, antibody, shANG, ANG specific short hairpin RNA, shCTRL, control short hairpin RNA, * *P* < 0.05. Scale bar = 50 μm

Active caspase 3 (AC-3) was used as a marker of apoptosis in IHC experiments, and representative images of AC-3 expression in each group are presented in Fig. 4a and c. Similar to our previous study, MSC co-transplantation significantly reduced follicular apoptosis in the Graft + MSC group compared with that in the Graft group in both the shRNA (12.3 ± 2.5% vs. 18.7 ± 3.3%, respectively, *P* < 0.05. Fig. 4b) and antibody blockade experiments (9.7 ± 3.4% vs. 20.0 ± 3.1%, respectively, *P* < 0.05. Fig. 4d). We hypothesized that ANG blockade may increase follicular apoptosis with co-transplantation of MSCs. Indeed, in the shRNA blockade experiment, shANG significantly increased follicular apoptosis, as a significantly elevated number of AC-3-positive primordial follicles was observed in the Graft + MSC shANG group (17.2 ± 2.4%) in comparison to the number in the Graft + MSC (12.3 ± 2.5%, *P* < 0.05. Fig 4b) and the Graft + shCTRL MSC groups (11.2 ± 2.0%, *P* < 0.05. Fig 4b). It is not surprising that shCTRL did not affect follicular apoptosis in xenografted ovarian tissues after the co-transplantation of MSCs, as the follicular apoptosis rate in the Graft + MSC shCTRL group (11.2 ± 2.0%) was not significantly different from that of the Graft + MSC group (12.3 ± 2.5%, *P* = 0.61, Fig. 4b).

In the antibody blockade experiment, the ANG antibody significantly increased follicular apoptosis, and significantly elevated numbers of AC-3-positive follicles were observed in the Graft + MSC + ANG Ab group (17.9 ± 2.1%) compared with the numbers in the Graft + MSC group (9.7 ± 3.4%, *P* < 0.05. Fig 4d) and the Graft + MSC + CTRL Ab group (11.5 ± 2.6%, *P* < 0.05. Fig 4d). It is not surprising that the CTRL IgG Ab did not affect follicular apoptosis in xenografted ovarian tissues after the co-transplantation of MSCs; the follicular apoptosis rate in the Graft + MSC + CTRL Ab group (11.5 ± 2.6%) was not significantly different from that of the Graft + MSC group (9.7 ± 3.4%, *P =* 0.47, Fig. 4d).

## Discussion

Our study highlights several important findings. The first is that the expression levels of a panel of proteins, including ANG, were significantly increased upon the co-transplantation of MSCs with ovarian tissues. The second is that the blockade of ANG using either the specific shANG or an ANG neutralizing antibody could suppress MSC-induced ovarian angiogenesis and follicle survival. These results indicated that MSC-derived ANG induced angiogenesis and follicle survival in xenografted human ovarian tissues. Our study is the first to demonstrate the crucial role of ANG in mediating the pro-angiogenic effect of MSCs co-transplanted with ovarian tissue, and these results provide a theoretical basis for further research on and the application of MSCs in ovarian tissue transplantation.

The ability of MSCs to release paracrine factors with pro-angiogenic functions has been observed in many studies. Umbilical cord MSC-derived microvesicles containing several angiogenic factors, including EGF and VEGF, contribute to the pro-angiogenic effect of MSCs [35]. Placental chorionic villus MSCs showing a high capacity to release angiogenic factors, including VEGF and hepatocyte growth factor, have been found to contribute to the functional improvement of ischemic hind limbs of nude mice after transplantation [36]. Pre-clinical and clinical studies have also shown that MSCs can augment cardiac function upon implantation into the ischemic/infarcted myocardium [37, 38]. These studies have suggested that pro-angiogenic factors released by MSCs could stimulate angiogenesis and increase regional perfusion. In our model of MSC and ovarian tissue co-transplantation, MSCs were packaged in Matrigel surrounding the ovarian graft, providing a suitable microenvironment for the release of pro-angiogenic proteins. Antibody-based cytokine array technology offers a novel approach for gaining insight into changes in protein expression profiles. Our array results reflected the secretion profiles of MSCs co-transplanted with ovarian tissues. Further analyses showed that despite the variety of angiogenic factors in the paracrine profiles of MSCs, the targeted inhibition of ANG had a substantial effect on the angiogenic ability of MSCs in ovarian transplantation. One possible explanation for this finding is that, as previously reported, the presence of ANG could be essential for other angiogenic factors, including VEGF and bFGF, to functionally induce angiogenesis [33].

ANG, also known as ribonuclease 5, is a secreted protein. In the past several years, studies have shown that ANG has angiogenic [39], neurogenic [40], and immune-regulatory [41] functions. Several pro-angiogenic factors, including ANG, have been isolated from the follicular fluid, and a positive association with follicular growth has also been suggested [42–45]. The present study suggests a critical role for ANG in establishing vascularization and promoting follicle survival in ovarian grafts co-transplanted with MSCs. It should be acknowledged that angiogenesis is a complicated process and that the pro-angiogenic effects of MSCs involve a variety of secreted factors. In our study, after the blockade of ANG, the pro-angiogenic effect of MSCs was attenuated. This inhibition was not complete, as the microvascular density after either shRNA or antibody blockade was still higher than in the Graft group; however, this difference did not reach statistical significance. This phenomenon might be explained by the fact that other factors independent of ANG may also be involved in the pro-angiogenic effect of MSCs. Interestingly, the array result suggests induced expression of both MMPs and TIMP-2 in MSCs transplanted ovary. MMPs were speculated to be induced by the hypoxic condition of ovarian grafts. Since TIMP-2 is an known antagonist of MMPs and endogenous inhibitor of angiogenesis, the induction of TIMP-2 may suppress excess formation of microvessels and non-functional microvessels. Additionally, it is worth noting that other secreted factors that have been reported to exhibit an anti-angiogenic capacity, such as angiopoietin-2, were significantly increased, as well, according to our microarray results. This result might be attributed to the equilibrium between angiogenic stimulators and inhibitors in the angiogenically balanced microenvironment established by the MSCs, which precisely regulates the rate of blood vessel formation.

Ischemia–reperfusion injury after ovarian tissue transplantation has been shown to lead to considerable follicular loss and to shorten the duration of ovarian function [46]. Graft angiogenesis following ovarian tissue transplantation is critical for graft viability. The re-establishment of vascularization in xenografted ovarian tissues is observed within 48 h of grafting, and this process lasts for approximately 1 week [47]. In an ovarian autograft study using mice, blood perfusion could be observed on day 3 after transplantation, and functional vessels could be detected on day 7 post-transplantation [48, 49]. Similarly, in a human study, microvessels started to form 48 h post-transplantation, whereas functional vessels required approximately 7 days to generate; thus, 7 days was proposed as an important landmark after transplantation [31, 50]. Therefore, in the present study, xenografted ovarian tissues were retrieved on day 7 post-transplantation. We selected the subcutaneous route for transplantation in this study since the subcutaneous areas of the abdominal wall have been reported to be one of the most preferable options for follicular development in ovarian transplantation [51, 52].

In recent years, numerous studies have focused on interventions to facilitate angiogenesis in transplanted ovarian tissue. Labied et al. [53] suggested that VEGF111 could stimulate vascular endothelial cell proliferation and functional angiogenesis, thereby increasing the viability of the ovarian cortex. Experimental data have also shown that basic fibroblast growth factor and fibrin hydrogel can improve angiogenesis and promote follicle development in mice [31]. However, none of these factors have been successfully utilized in a clinical setting thus far. Further investigation of methods for improving angiogenesis in ovarian tissue transplantation is required. Our previous study showed that MSCs show promise for the improvement of ovarian tissue transplantation and follicle growth [13, 26]. Although MSCs can be obtained from bone marrow, readily cultured and preserved for future use, further research is needed to develop methods for their use. Our findings suggest mechanistic explanations for the angiogenic effect of MSCs in ovarian transplantation, providing important information for further applications.

Previous studies have indicated that the therapeutic application of secreted molecules as a possible replacement for stem cells might lead to the development of safe and effective therapeutic strategies with predictable outcomes [22]. Therefore, practical interventions to facilitate angiogenesis, including treatment with exogenous angiogenic factors, might be promising in clinical settings, as this approach will circumvent the ethical and safety constraints associated with the direct use of human MSCs. In our preliminary study, the treatment of xenografted ovarian tissues with exogenous recombinant ANG enhanced angiogenesis, even without MSCs (data not shown). However, due to the scarcity of human ovarian tissue samples available for research, we were unable to comprehensively investigate the outcomes of ovarian transplantation through treatment with exogenous recombinant ANG protein. Additional studies of the use of human ovarian cortical grafts with long-term ANG treatment are necessary to elucidate the effects and functional mechanism of ANG in mediating the angiogenesis of xenografted ovarian tissue. Continuing research on this topic will help elucidate the efficacy and safety of exogenous ANG intervention in ovarian tissue transplantation in clinical settings.

## Conclusions

In conclusion, our findings are the first to suggest that MSCs mediate angiogenesis and follicle survival in xenografted human ovarian tissue through ANG. Our results provide a theoretical basis and important information for the further application of MSCs in ovarian tissue transplantation and could eventually aid patients in preserving fertility.

## Additional files

Additional file 1: Figure S1. Isolation of MSCs from human bone marrow tissues by density gradient centrifugation. (JPG 727 kb)
Additional file 2: Figure S2. Identification of MSCs by Flow Cytometry. (A) Representative histogram of FACS results showing the MSCs surface marker profile. The blue peak indicated the specific antibodies: CD34, CD45, CD19, negative cocktail (including CD44, CD90 and CD105). The red peak represented the isotope antibodies. (B) Positive expression of CD34, CD45, CD19 and negative cocktail (including CD44, CD90 and CD105) in five individuals as examined by flow cytometry was expressed as mean ± SD. The proportion of cells expressing CD44, CD90, CD105 and negative cocktail, which were analyzed from 5 independent samples, were 96.6% ± 2.1%, 96.4% ± 2.2%, 97.2% ± 2.0% and 2.9% ± 0.6%. (JPG 418 kb)
Additional file 3: Figure S3. Representative images showing expression of CD90 and CD105 in ovarian sections after co-transplantation of MSCs in both high and low magnification. MSC, mesenchymal stem cells. Scale bar = 200 μm in A and C, Scale bar = 50 μm in B and D. (JPG 453 kb)
Additional file 4: Figure S4. The MSC clones stably knocking down ANG were identified and verified on qPCR and ELISA analysis. A) Results of quantitative PCR showed a significant knock-down of ANG mRNA expression in the shANG transfected MSCs (n = 3). B) Secreted ANG protein level in the shANG and shCTRL transfected MSCs groups were determined by ELISA (n = 3). Data are shown as means ± SEM of triplicates in a representative experiment. MSC, mesenchymal stem cells; ANG, angiogenin; shANG, ANG specific short hairpin RNA, shCTRL: control short hairpin RNA, * *P* < 0.01. (TIF 7881 kb)
Additional file 5: Figure S5. Representative images showing triple staining of Ki67, DAPI and CD31 in ovarian graft with or without co-transplantation of MSCs. Vasculature is shown in red, cell nuclei are shown in blue and Ki67 positive nuclei are shown in green. Scale bar = 50 μm. (JPG 299 kb)
Additional file 6: Figure S6. Representative images showing HE staining of ovarian sections in ovarian graft with or without co-transplantation of MSCs in both high and low magnification. MSC, mesenchymal stem cells. Scale bar = 200 μm in A and C, Scale bar = 50 μm in B and D. (JPG 507 kb)

## Abbreviations

Ab: Antibody
ANG: Angiogenin
CTRL: control
MSC: Mesenchymal stem cells
shANG: ANG specific short hairpin RNA
shCTRL: Control short hairpin RNA
shRNA: Short hairpin RNA
