# Supplementary figures and images for: Mesenchymal stem cell-derived angiogenin promotes primodial follicle survival and angiogenesis in transplanted human ovarian tissue
Source: Reprod Biol Endocrinol. 2017 Mar 9;15:18. doi: 10.1186/s12958-017-0235-8 (PMC5343383; doi:10.1186/s12958-017-0235-8)

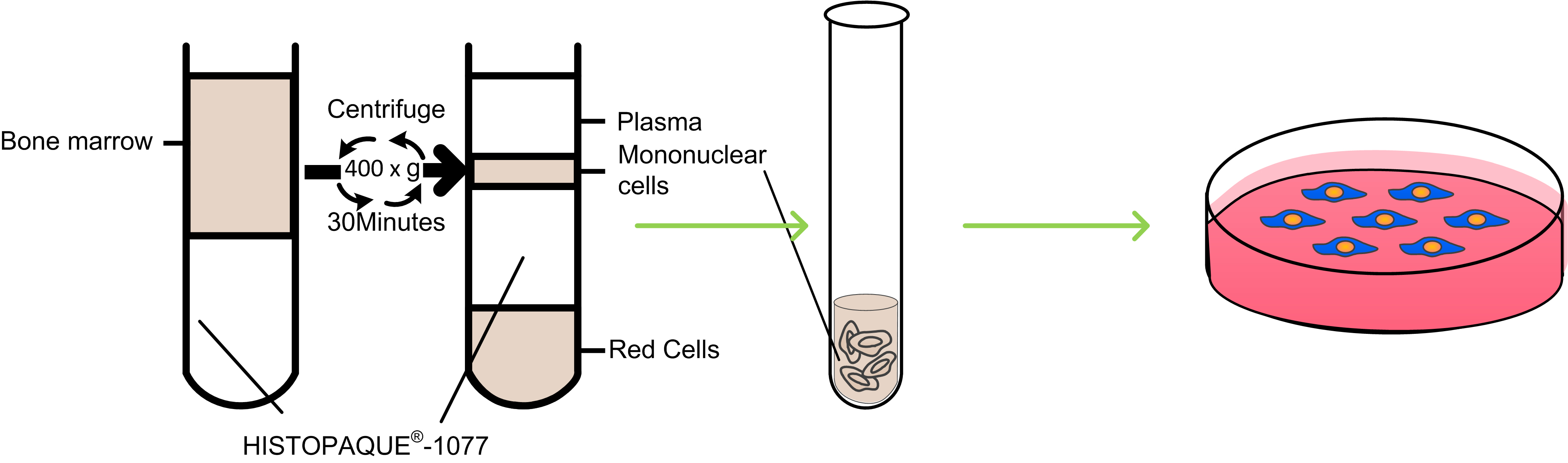

Supplement: Additional file 1: Figure S1. — Isolation of MSCs from human bone marrow tissues by density gradient centrifugation. (JPG 727 kb) [file 12958_2017_235_MOESM1_ESM.jpg]

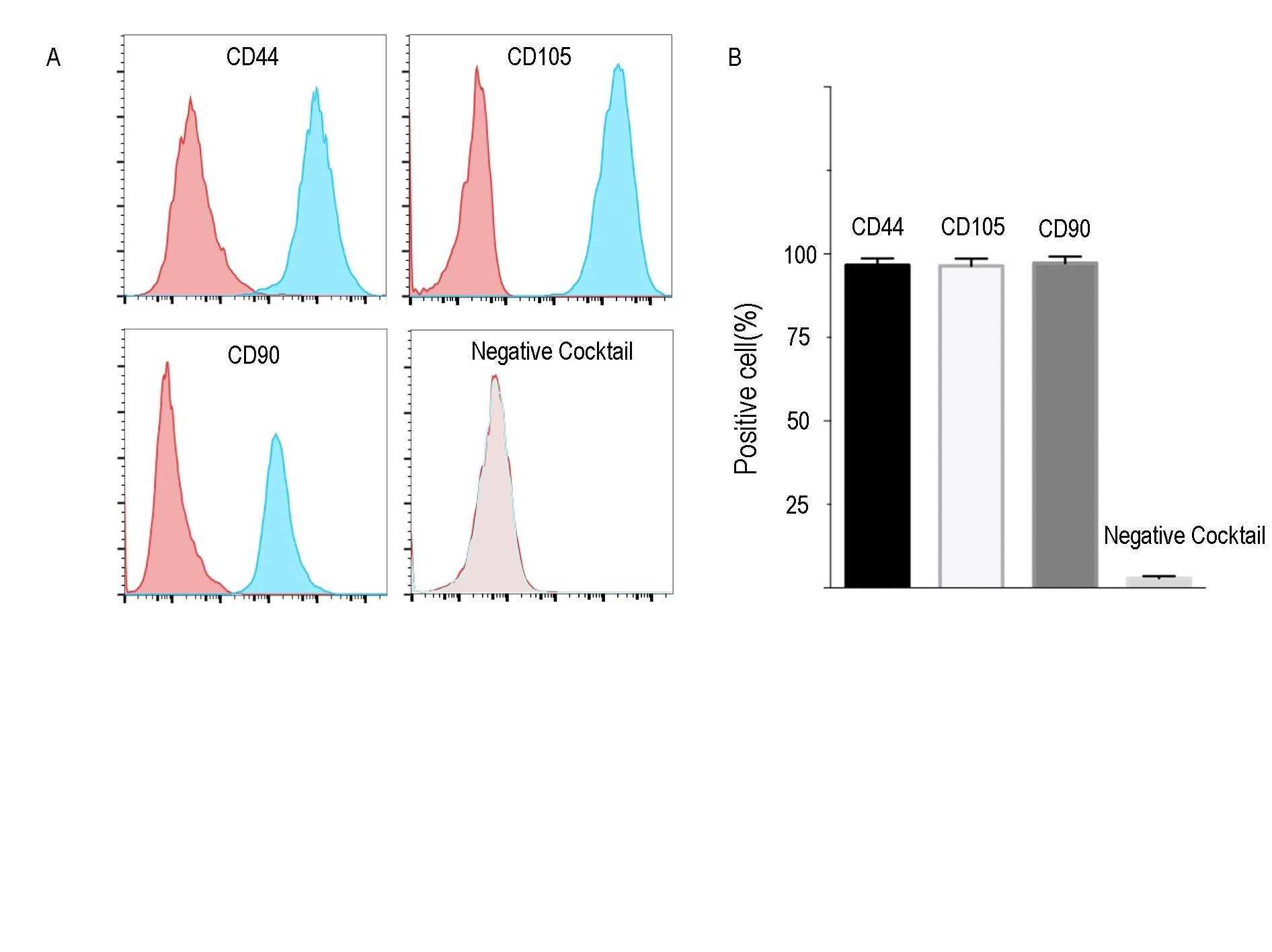

Supplement: Additional file 2: Figure S2. — Identification of MSCs by Flow Cytometry. (A) Representative histogram of FACS results showing the MSCs surface marker profile. The blue peak indicated the specific antibodies: CD34, CD45, CD19, negative cocktail (including CD44, CD90 and CD105). The red peak represented the isotope antibodies. (B) Positive expression of CD34, CD45, CD19 and negative cocktail (including CD44, CD90 and CD105) in five individuals as examined by flow cytometry was expressed as mean ± SD. The proportion of cells expressing CD44, CD90, CD105 and negative cocktail, which were analyzed from 5 independent samples, were 96.6% ± 2.1%, 96.4% ± 2.2%, 97.2% ± 2.0% and 2.9% ± 0.6%. (JPG 418 kb) [file 12958_2017_235_MOESM2_ESM.jpg]

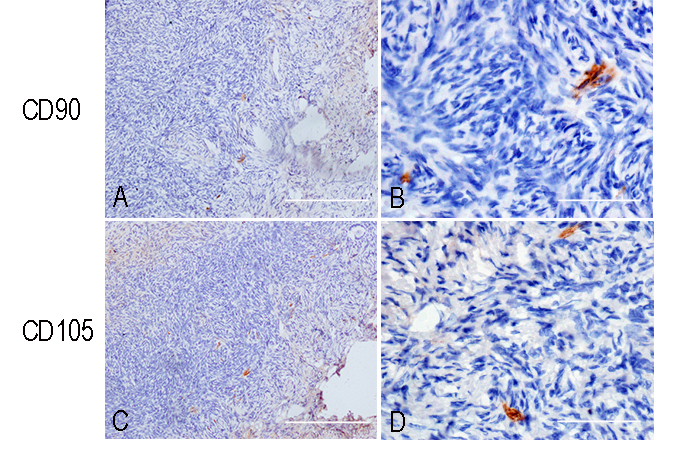

Supplement: Additional file 3: Figure S3. — Representative images showing expression of CD90 and CD105 in ovarian sections after co-transplantation of MSCs in both high and low magnification. MSC, mesenchymal stem cells. Scale bar = 200 μm in A and C, Scale bar = 50 μm in B and D. (JPG 453 kb) [file 12958_2017_235_MOESM3_ESM.jpg]

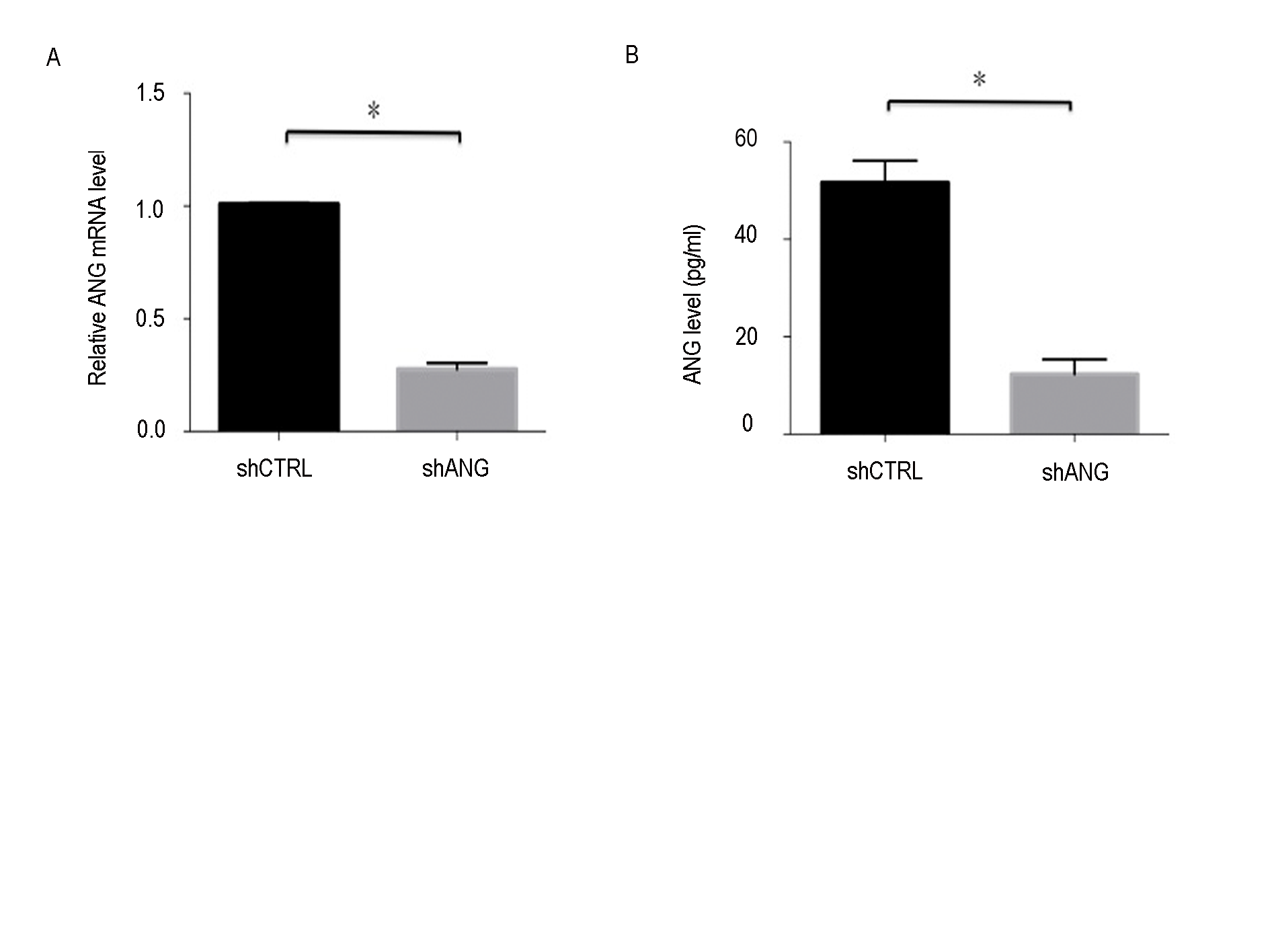

Supplement: Additional file 4: Figure S4. — The MSC clones stably knocking down ANG were identified and verified on qPCR and ELISA analysis. A) Results of quantitative PCR showed a significant knock-down of ANG mRNA expression in the shANG transfected MSCs (n = 3). B) Secreted ANG protein level in the shANG and shCTRL transfected MSCs groups were determined by ELISA (n = 3). Data are shown as means ± SEM of triplicates in a representative experiment. MSC, mesenchymal stem cells; ANG, angiogenin; shANG, ANG specific short hairpin RNA, shCTRL: control short hairpin RNA, * P < 0.01. (TIF 7881 kb) [file 12958_2017_235_MOESM4_ESM.tif]

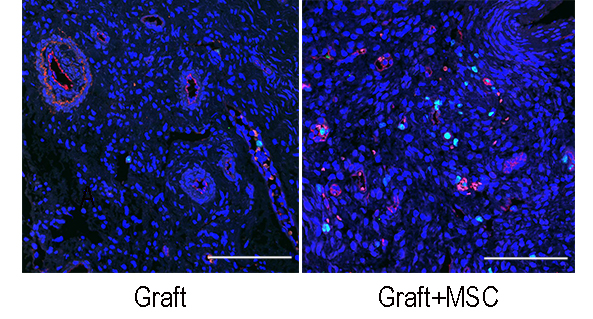

Supplement: Additional file 5: Figure S5. — Representative images showing triple staining of Ki67, DAPI and CD31 in ovarian graft with or without co-transplantation of MSCs. Vasculature is shown in red, cell nuclei are shown in blue and Ki67 positive nuclei are shown in green. Scale bar = 50 μm. (JPG 299 kb) [file 12958_2017_235_MOESM5_ESM.jpg]

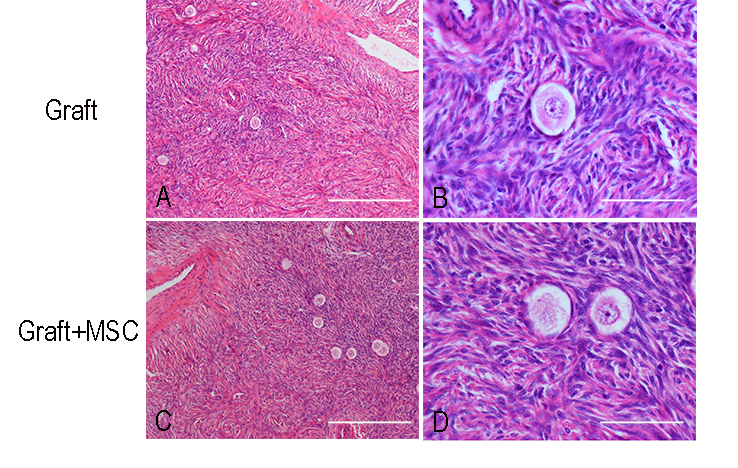

Supplement: Additional file 6: Figure S6. — Representative images showing HE staining of ovarian sections in ovarian graft with or without co-transplantation of MSCs in both high and low magnification. MSC, mesenchymal stem cells. Scale bar = 200 μm in A and C, Scale bar = 50 μm in B and D. (JPG 507 kb) [file 12958_2017_235_MOESM6_ESM.jpg]
